# Differential Fibroblast Growth Factor 8 (FGF8)-Mediated Autoregulation of Its Cognate Receptors, *Fgfr1* and *Fgfr3*, in Neuronal Cell Lines

**DOI:** 10.1371/journal.pone.0010143

**Published:** 2010-04-12

**Authors:** Natasha N. Mott, Wilson C. J. Chung, Pei-San Tsai, Toni R. Pak

**Affiliations:** 1 Department of Cell and Molecular Physiology, Loyola University Chicago Stritch School of Medicine, Maywood, Illinois, United States of America; 2 Department of Integrative Physiology, University of Colorado at Boulder, Boulder, Colorado, United States of America; Universidade Federal do Rio de Janeiro (UFRJ), Brazil

## Abstract

Fibroblast growth factors (FGFs) mediate a vast range of CNS developmental processes including neural induction, proliferation, migration, and cell survival. Despite the critical role of FGF signaling for normal CNS development, few reports describe the mechanisms that regulate FGF receptor gene expression in the brain. We tested whether FGF8 could autoregulate two of its cognate receptors, *Fgfr1* and *Fgfr3*, in three murine cell lines with different lineages: fibroblast-derived cells (3T3 cells), neuronal cells derived from hippocampus (HT-22 cells), and neuroendocrine cells derived from hypothalamic gonadotropin-releasing hormone (GnRH) neurons (GT1-7 cells). GnRH is produced by neurons in the hypothalamus and is absolutely required for reproductive competence in vertebrate animals. Several lines of evidence strongly suggest that *Fgf8* is critical for normal development of the GnRH system, therefore, the GT1-7 cells provided us with an additional endpoint, *Gnrh* gene expression and promoter activity, to assess potential downstream consequences of FGF8-induced modulation of FGF receptor levels. Results from this study suggest that the autoregulation of its cognate receptor represents a common downstream effect of FGF8. Further, we show that *Fgfr1* and *Fgfr3* are differentially regulated within the same cell type, implicating these two receptors in different biological roles. Moreover, *Fgfr1* and *Fgfr3* are differentially regulated among different cell types, suggesting such autoregulation occurs in a cell type-specific fashion. Lastly, we demonstrate that FGF8b decreases *Gnrh* promoter activity and gene expression, possibly reflecting a downstream consequence of altered FGF receptor populations. Together, our data bring forth the possibility that, in addition to the FGF synexpression group, autoregulation of FGFR expression by FGF8 represents a mechanism by which FGF8 could fine-tune its regulatory actions.

## Introduction

Fibroblast growth factors (FGFs) mediate a vast range of CNS developmental processes including neural induction, proliferation, migration, and cell survival. The FGF family consists of four receptors (FGFR1, 2, 3, 4), 22 ligands, and their splice variants that vary in expression patterns both temporally and spatially [1]. The structural components of FGF receptors consist of three extracellular Ig-like domains, a transmembrane domain, and two intracellular tyrosine kinase domains [2]. Despite the critical role of FGF signaling in CNS development, there are few reports to date describing the mechanisms that regulate FGF receptor gene expression in the brain.

Receptor expression is often controlled by autoregulation, where binding of the cognate ligand leads to changes that affect protein turnover, internalization, primary transcript stability, and gene promoter activity [3], [4], [5]. Interestingly, FGFR1 was reported to have a synexpression pattern with its cognate ligand FGF8 [6]. Synexpression is an interesting feature associated with FGF and a few other signaling pathways that involves the coexpression of a set of genes termed the synexpression group [7], [8], [9]. The products of the FGF synexpression group are then capable of modulating the intracellular signaling cascades of several FGF ligands, in particular FGF8, to curtail or achieve specific spatial patterns of FGF signaling [10]. This raises the possibility that FGF8 may control its own activity level via the autoregulation of its own receptors. The upregulation of FGFR1 by FGF8 could represent a positive feedback mechanism that adds another layer of regulatory complexity, further fine-tuning the spatial and temporal specificity of FGF8 actions during development.

Until now, the possibility that FGF8 could add to the modular regulation of its activity in neurons by autoregulating its own receptor has not been adequately explored. Further, it is unclear if FGF8 could autoregulate all cognate receptors in a similar fashion. In this study, we examined if FGF8 autoregulated two of its cognate receptors, *Fgfr1* and *Fgfr3*, in three murine cell lines with different lineages: fibroblast-derived cells (3T3), neuronal cells derived from hippocampus (HT-22), and neuroendocrine cells derived from hypothalamic gonadotropin-releasing hormone neurons (GT1-7). The GT1-7 cells were particularly useful since the *in vivo* specification of GnRH neuronal fate was shown to be highly dependent on FGF8 signaling and, the expression level of FGF receptors in these cells could be correlated with a hallmark of GnRH neuronal differentiation: the expression of *Gnrh* gene [11]. Therefore, these cells provided us with an additional endpoint, *Gnrh* gene expression and promoter activity, to assess potential downstream consequences of FGF8-induced modulation of FGF receptor levels.

## Results

### Endogenous expression of FGF8 in 3T3, HT-22, and GT1-7 cell lines

**Figure 1:**
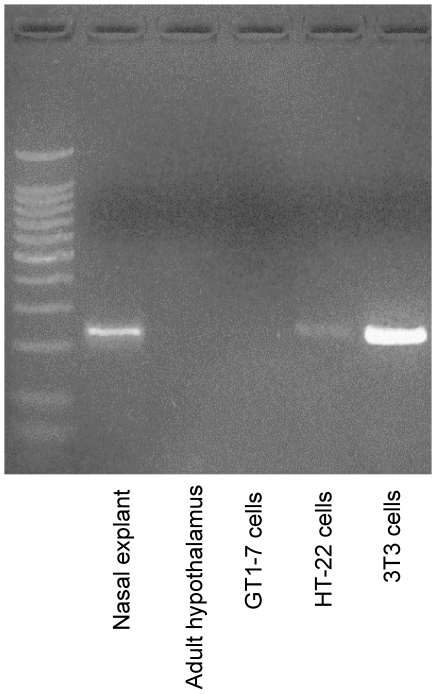
FGF expression in mouse brain and representative cell lines. Photomicrograph of RT-PCR product for FGF8 mRNA stained with ethidium bromide and resolved on a 2% agarose gel. Total RNA was isolated from mouse nasal explant (embryonic day 11.5; lane 2) adult hypothalamus (lane 3), hypothalamic-derived GT1-7 cells (lane 4), hippocampus-derived HT-22 cells (lane 5), and fibroblast-derived 3T3 cells (lane 6). Presence of band indicates FGF8 primary transcripts in representative tissue type or cell line.

First, we characterized the endogenous expression of FGF8 in the 3T3, HT-22, and GT1-7 and compared it with mouse tissue taken from embryonic nasal explants and adult hypothalamus. Consistent with the widely accepted role of FGF8 during development, mouse nasal explants had high expression levels of endogenous FGF8 (Fig. 1, lane 2). Also, 3T3 cells had high endogenous levels of FGF8 (Fig. 1, lane 6) which was expected due to their fibroblast cell lineage. By contrast, endogenous FGF8 expression was low in the neuronal-derived HT-22 cells (Fig. 1, lane 5) and completely absent in the GT1-7 cells and hypothalamus (Fig. 1, lanes 3 and 4, respectively).

### Differential effects of FGF8b on the expression of FGF receptors 1 (*Fgfr1*) and 3 (*Fgfr3*)

**Figure 2:**
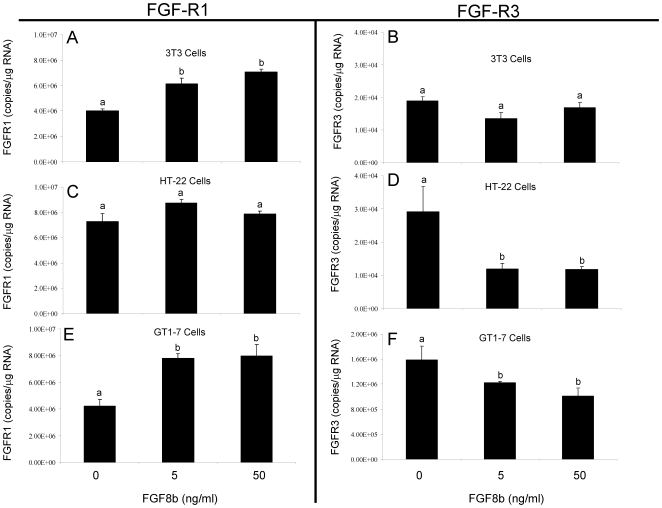
Comparative effects of FGF8b on *Fgfr1* and *Fgfr3* mRNA in 3T3, HT-22 and GT1-7 cells. *Fgfr1* and *Fgfr3* mRNA levels in 3T3 cells (A, B), HT-22 cells (C, D), or GT1-7 cells (E,F) following treatment with vehicle or FGF8b at 5 or 50 ng/ml. for 4 hours. Data are expressed as mean copies of *Fgfr1* or *Fgfr3* transcript/µg total RNA ± SEM. Dissimilar letters indicate statistically significant difference among groups, P<0.05.

In these experiments, 3T3, HT-22, and GT1-7 cells were treated with 5 or 50 ng/ml of FGF8b for 4 hours in order to determine if FGF8b regulated the expression of its cognate receptors *Fgfr1* and *Fgfr3* in these cell types. Overall, our data revealed that FGF8b differentially altered the expression of *Fgfr1* and *Fgfr3* mRNA depending on the cell type. For instance, in 3T3 cells, which express high endogenous levels of FGF8, FGF8b treatment for 4 hours significantly increased *Fgfr1* mRNA (Fig. 2A), yet had no effect on *Fgfr3* (Fig. 2B). By contrast, in HT-22 cells, which express low endogenous levels of FGF8, FGF8b treatment had no effect on *Fgfr1* (Fig. 2C), yet significantly decreased *Fgfr3* mRNA (Fig. 2D). Most notably, in the GT1-7 cells, which do not express endogenous FGF8, FGF8b treatment significantly increased *Fgfr1* expression (Fig. 2E) while simultaneously decreasing *Fgfr3* expression (Fig. 2F). This differential effect of FGF8b on *Fgfr1* and *Fgfr3* receptor expression in GT1-7 cells did not occur in the HT-22 or 3T3 cells. Therefore, in a subsequent experiment, GT1-7 cells were used to determine whether the differential effects of FGF8b on *Fgfr1* and *Fgfr3* expression are mediated through the classical membrane FGF receptors.

### FGF8b effects on FGF receptor expression in GT1-7 cells are mediated by FGF receptors

**Figure 3:**
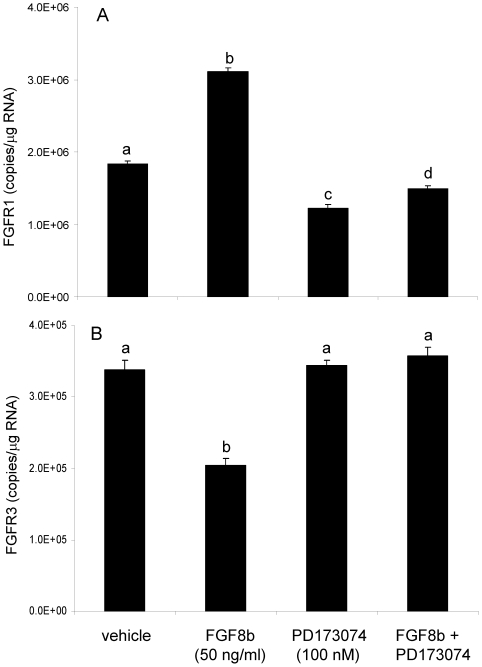
Differential effects of FGF8b on *Fgfr1* and *Fgfr3* mRNA in GT1-7 cells. *Fgfr1* (A) and *Fgfr3* (B) mRNA levels in GT1-7 cells following treatment with vehicle, FGF8b (50 ng/ml), PD173074 (100 nM), or FGF8b + PD173074 for 8 hours. Data are expressed as mean copies of *Fgfr1* or *Fgfr3*/µg total RNA ± SEM. Dissimilar letters indicate statistically significant difference among groups, P<0.05.

In these experiments, GT1-7 cells were treated with FGF8b (50 ng/ml), the FGF receptor antagonist PD173074 (100 nM), or combined FGF8b + PD173074 for 8 hours. Consistent with our earlier observation, treatment with FGF8b alone significantly increased *Fgfr1* (Fig. 3A). Moreover, the FGF receptor anatagonist PD173074, alone or in combination with FGF8b, significantly inhibited *Fgfr1* mRNA expression compared to the vehicle group (Fig. 3A). Also consistent with our earlier observation in GT1-7 cells, FGF8b treatment significantly decreased *Fgfr3* mRNA (Fig. 3B), but there was no effect of PD173074 alone on the expression of *Fgfr3*. The inhibitory effect of FGF8b on *Fgfr3* was completely abolished in the presence of the antagonist (Fig. 3B).

### FGF8b decreased gonadotropin-releasing hormone promoter activity and mRNA in GT1-7 cells

Previous work demonstrated that targeted disruption of *Fgfr1* signaling in GnRH neurons decreased the numbers of detectable GnRH neurons in the hypothalamus of adult mouse brain, suggesting that FGF is critical for normal GnRH neuronal development [12] Based on this observation, we hypothesized that that the expression level of the *Gnrh* gene, a hallmark of GnRH neuronal differentiation, could vary according to *Fgfr1*, and possibly *Fgfr3*, levels [11]. Further, in the previous experiment we determined that FGF8b differentially altered the expression of its two cognate receptors, *Fgfr1* and *Fgfr3* in a GnRH-expressing cell line (GT1-7 cells; see Fig. 2E, F). Therefore, we measured *Gnrh* promoter activity and mRNA levels in GT1-7 cells following treatment with FGF8b to determine whether changes in the FGF receptor population (i.e increased *Fgfr1* and decreased *Fgfr3*) corresponded to changes in *Gnrh* gene activity.

**Figure 4:**
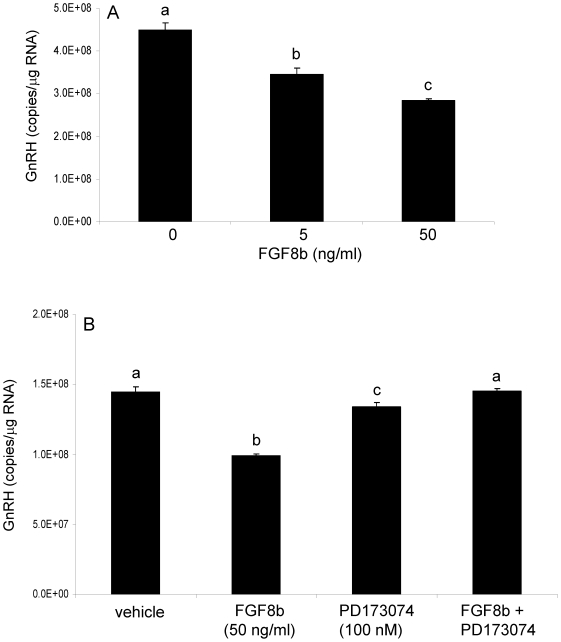
FGF8b decreased GnRH mRNA in GT1-7 cells. Panel A: GnRH mRNA levels in GT1-7 cells following 8 hours of vehicle or FGF8b treatment at 5 or 50 ng/ml. Panel B: GnRH mRNA levels in GT1-7 cells following treatment with vehicle, FGF8b (50 ng/ml), PD173074 (100 nM), or FGF8b + PD173074 for 8 hours. Data are expressed as mean copies of GnRH transcript/µg total RNA ± SEM. Dissimilar letters indicate statistically significant difference among groups, P<0.05.

**Figure 5:**
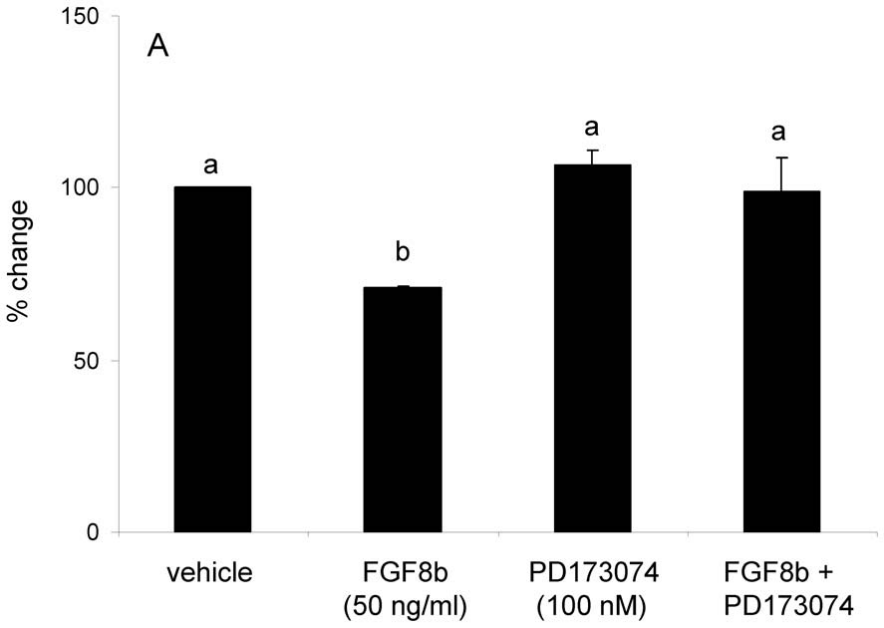
Effects of FGF8b on GnRH promoter activity. Transient transfection of GT1-7 cells with 0.15 µg/well of mouse full-length GnRH-luciferase reporter construct. Following transfection, cells were treated with vehicle, FGF8b (50 ng/ml), PD173074 (100 nM), or FGF8b + PD173074 for 8 hours. Data are represented as mean percent change in RLU's from vehicle-treated controls ± SEM. Dissimilar letters indicate statistically significant difference among groups, P<0.05.

GT1-7 cells were treated with 5 or 50 ng/ml of FGF8b for 4 or 8 hours. Treatment with either 5 or 50 ng/ml of FGF8b significantly reduced *Gnrh* mRNA in GT1-7 cells after 8, but not 4 (data not shown), hours of FGF8b exposure (Fig. 4A). To determine whether the effects of FGF8b on *Gnrh* expression in GT1-7 cells were mediated through classical FGF receptors, cells were treated with the broad FGF receptor antagonist PD173074. As expected, FGF8b treatment concomitant with the receptor antagonist had no effect on *Gnrh* mRNA levels (Fig. 4B), indicating that the FGF8b-induced decrease in *Gnrh* mRNA in GT1-7 cells was dependent upon its cognate membrane receptors. Interestingly, treatment with PD173074 alone induced a modest, yet significant, decrease in *Gnrh* mRNA levels, similar that observed previously with *Fgfr1* (compare to Fig. 3A). Next, we measured *Gnrh* promoter activity following FGF8b treatment in GT1-7 cells. Treatment with FGF8b for 8 hours significantly reduced *Gnrh* promoter activity in GT1-7 cells (Fig. 5) in parallel to the observed reduction in *Gnrh* mRNA levels (compare to Fig. 4A). Further, the concomitant treatment with PD17074 abolished the FGF8b-induced reduction in promoter activity in GT1-7 cells.

## Discussion

Precisely timed and coordinated FGF signaling events are critical for proper CNS development, yet the mechanisms regulating the expression of specific membrane FGF receptors in neurons have not been thoroughly investigated. From our data, the following general conclusions can be drawn. First, FGF8b autoregulates the gene expression of its two cognate receptors, *Fgfr1* and *Fgfr3*, in a cell-type specific manner; second, this autoregulation is mediated by FGF receptors as opposed to a non-classical signaling pathway; third, receptor specific autoregulation might be dependent upon the levels of endogenous FGF8 present in a given cell type; and finally, FGF8b decreases *Gnrh* promoter activity and gene expression, possibly reflecting a downstream consequence of altered FGF receptor populations.

Ligand-mediated receptor autoregulation is a common feature of many types of receptors, but there are few reports documenting this as a mechanism for regulating FGF receptor gene expression. FGF receptors belong to a large class of cell surface receptors called receptor tyrosine kinases (RTKs) which encompasses multiple receptor families, including the epidermal growth factor (EGF), vascular endothelial growth factor (VEGF), RET, and Eph receptor families [1], [2], [13]. The initial reports characterizing the discovery of an FGF receptor noted that basic FGF (FGF2) downregulated the number of available FGF binding sites in baby hamster kidney cells [14]. These results were later confirmed in the fibroblast-derived 3T3 cell line [15] and in pancreatic-derived AR4-2J cells [16]. Interestingly, while FGF2 downregulated *Fgfr1* in AR4-2J cells, an FGF2 protein isoform (22.5 kDa FGF2) upregulated *Fgfr1* levels in those same cells by increasing the half-life of the *Fgfr1* transcript [16]. For our studies, FGF8b, was chosen over other FGF8 isoforms based on the greater requirement of FGF8b during CNS development [17], [18]. In this respect, the earlier findings are consistent with our current data in supporting the autoregulatory effects of an FGF ligand on its own receptors, at the level of transcription.

The four FGF receptors and their associated splice variants share a considerable amount of overlap in their tissue distribution, ability to bind multiple FGF ligands, and their intracellular signaling pathways. Despite this redundancy, there is mounting evidence that each receptor confers distinct downstream cellular functions. Studies using the tumorigenic pro-B cell line, BaF3, showed that a majority of the FGF ligands are more effective at inducing mitogenic activity through *Fgfr1* than the other FGF receptor types [1]. Moreover, abnormally high levels of *Fgfr1* gene expression have been observed in prostate, colorectal, bladder, and a subset of breast carcinomas [13], [19], [20], [21]. Together, these studies suggest that *Fgfr1* is important for maintaining or inducing a less differentiated cell phenotype. On the other hand, it has been shown that *Fgfr3* can inhibit cellular proliferation in multiple cell types including bone, pancreas, and brain [22], [23], [24], [25]. Further, a reciprocal relationship between *Fgfr1* and *Fgfr3* was observed in colorectal carcinoma cells [20]. In that study, the transcriptional silencing of *Fgfr1* with siRNA decreased cellular proliferation and increased *Fgfr3* expression, suggesting that *Fgfr3* was important for limiting the progression of tumorigenesis. The only cell line in the present study that recapitulated the reciprocal relationship between *Fgfr1* and *Fgfr3* was GT1-7 cells (Fig. 2). As such, we hypothesize that *Fgfr1* and *Fgfr3* both mediate biologically important, but opposing, effects in these cells. FGF8 could favor effects mediated by *Fgfr1* by upregulating *Fgfr1* and downregulating the antagonizing *Fgfr3*. These data put forth a novel mechanism by which a ligand with multiple receptors could preferentially activate pathways associated with one receptor subtype. This regulatory mechanism would offer both flexibility and selectivity during development, when multiple ligands and their receptors are present at the same time.

Strong evidence suggests that FGF8 signaling, through its cognate receptor *Fgfr1*, is critical for normal development of the GnRH system. Mice hypomorphic for *Fgf8* or *Fgfr1* possessed virtually no GnRH neurons in their forebrains [11]. By contrast, *Fgfr3*-null mice showed no developmental deficiencies in GnRH neurons (13). In this respect, *Fgfr1* and *Fgfr3* are clearly not functionally equivalent in driving GnRH neuronal system development, although both are expressed in GnRH neurons [26]. In GT1-7 cells treated with FGF8b, an increase in *Fgfr1*/*Fgfr3* ratio was accompanied by a concomitant decrease in *Gnrh* mRNA. Extrapolating these results to the endogenous GnRH neurons, FGF8b could induce a general suppression of *Gnrh* gene expression during early development via the preferential activation of *Fgfr1*, a phenomenon consistent with low levels of *Gnrh* gene expression in GnRH neurons before birth [27]. However, the physiological significance of this finding in the endogenous GnRH system requires further exploration.

Overall, our data demonstrate that autoregulation of FGFRs is a cell-type specific process that leads to altered downstream consequences due to individual receptor signaling events. At present, the molecular mechanisms regulating FGF ligand-induced receptor autoregulation and the resulting downstream effects are unclear. However, the data herein provide novel insights into understanding how FGF signaling, with 22 ligands, 4 transmembrane receptors, and their splice variants, could fine-tune their regulatory roles by differentially autoregulating FGF receptor transcription. Such a mechanism could be broadly applicable to the regulation of normal cellular processes, such as neural development, as well as pathological processes, such as cancer.

## Materials and Methods

### Cell culture

All cell lines used in these studies were verified to be free of mycoplasma contamination (MycoSensor QPCR, Stratagene/Agilent Technologies). The following murine tumorigenic cell lines were used: fibroblast-derived (3T3, American Tissue Type Culture Collection), neuronal derived from hippocampus (HT-22, a subclone of the HT4 cell line [28], generously provided by Dr. Dave Schubert, Salk Institute, San Diego, CA), and neuroendocrine derived from hypothalamic gonadotropin-releasing hormone neurons (GT1-7, generously provided by Dr. Pamela Mellon, University of California, San Diego, CA). Cells were maintained in 50/50 F12/Dulbecco's modified essential medium (DMEM) containing 4.5% glucose and L-glutamine (Invitrogen Inc., Carlsbad, CA), supplemented with 1x non-essential amino acids and 10% fetal bovine serum (Gemini Bioproducts, Woodland, CA). Cells were grown to 70% confluency and used within 10 passages for all experiments.

### Peptides

Recombinant mouse fibroblast growth factor-8b carrier-free (FGF8b, R&D Systems, Minneapolis, MN) was reconstituted in sterile phosphate buffered saline (PBS) and diluted further to final concentrations. The FGF/VEGF receptor tyrosine kinase inhibitor PD173074 (Calbiochem, Gibbstown, NJ) was reconstituted in 100% dimethlysulfoxide (DMSO) and used at a final concentration of 100 nM in 0.01% DMSO. PD173074 is an ATP-competitive reversible inhibitor of FGF and VEGF receptors (IC50  = 21.5 nM for FGFR1, Calbiochem) and has been extensively characterized [29], [30], [31], [32].

### Reporter plasmid constructs

The full-length mouse (-3446 to +24) GnRH promoter was subcloned into the promoterless firefly luciferase vector (pXP2) and has been extensively characterized [33], [34]. The renilla luciferase pGL4 reporter construct (Invitrogen Inc., Carlsbad, CA) was used as an internal control for calculating plasmid transfection efficiency.

### Transient Transfections

GT1-7 cells were plated at a density of 0.2×10^5^ cells/well in 96-well plates for 48 hours prior to transfection to achieve a final confluency of 70–80%. All constructs were transfected in replicates of six wells within each assay, and each transfection assay was repeated a minimum of 3 times. Further, each experiment was performed using a minimum of 3 different preparations for each plasmid reporter construct. Transfections were carried out using a lipid-mediated transfection reagent according to manufacturer's instructions (Fugene6, Roche Molecular Biomedical, Indianapolis, IN). Cells were incubated with transfection media complex overnight followed by replacement with phenol red-free 50/50 F12/DMEM containing 1.0% stripped fetal bovine serum (Hyclone Laboratories, Logan, UT) to minimize the presence of exogenous growth factors in the cell culture media. Notably, all experiments were replicated using media containing 10%, 1%, and dextran-charcoal stripped serum, and no differences were observed. Therefore, all data reported herein are taken from experiments where cells were kept in media containing 1% FBS. Thirty-six hours after transfection, cells were incubated with media containing 0.01% PBS, 50 ng/ml FGF8b, or 100 nM PD173074 for eight hours and then lysed for dual luciferase analysis. Luciferase activity was measured using the Dual Luciferase Reporter Assay system (Promega Corp., Madison, WI) according to manufacturer's instructions. Relative light units were measured using the Synergy HT multimode plate reader (BioTek Instruments Corp., Winooski, VT). Luciferase substrates (100 µl/well) were added to cells using automatic injectors attached to the plate reader.

### RNA isolation

3T3, HT-22, and GT1-7 cells were plated at a density of 2.0×10^5^ cells/well in a six-well plate. Cells were allowed to grow in regular media containing 10% FBS for 24–48 hours until 70–80% confluent. Twenty-four hours prior to treatment, cells were washed once with PBS followed by the addition of phenol red-free 50/50 F12/DMEM containing 1% FBS (Hyclone Laboratories, Logan, UT) to minimize the presence of exogenous growth factors in the cell culture media. Notably, all experiments were replicated using media containing 10%, 1%, and dextran-charcoal stripped serum and no differences were observed. All data reported herein are from cells kept in media containing 1% FBS. On the day of treatment, cells were treated with vehicle, 5 or 50 ng/ml FGF8b, 100 nM PD173074, or a combination (FGF8b + PD173074) for 4 or 8 hours. All treatments were done in replicates of 6 wells. Cells were washed once with cold PBS, lysed with Trizol reagent, and total RNA isolated according to manufacturer's instructions (Invitrogen Inc., Carlsbad, CA). Following isolation, genomic DNA contamination was removed using DNAfree reagents (Stratagene, a division of Agilent Corp., La Jolla, CA) according to manufacturer's instructions. Quantification of total RNA was performed using a Nanodrop spectrophotometer, and samples with an OD 260∶280 of 1.7 – 1.9 were used for subsequent reverse transcription assays.

### Reverse Transcription

Total RNA (1 µg from cell culture experiments for quantitative real-time RT-PCR; 0.5 µg for FGF8 detection RT-PCR in embryonic nasal explants at E11.5, adult hypothalamus, 3T3 cells, HT-22 cells, and GT1-7 cells) was combined with 0.5 µg oligo d(T), heated to 65°C and rapidly cooled on ice. The RNA-primer mix was combined with M-MLV buffer (50 mM Tris-HCl pH 8.3, 75 mM KCl, 3 mM MgCl_2_), 10 mM DTT, 0.5 mM dNTP and 0.5 mM M-MLV reverse transcriptase (Invitrogen Inc., Carlsbad, CA). Reverse transcriptase reaction was performed by incubating for 10 minutes at room temp, 50 minutes at 42°C, then 95°C for 5 minutes to terminate the reaction.

### RT-PCR

FGF8 Detection. 2 µl of cDNA template, prepared by reverse transcription reaction as described above, was added to a master mix containing 1x Go Taq flexi buffer (Promega Corp.), 1.5 mM MgCl, 200 µM dNTP mix, 0.5 µM forward and reverse primer (see primer sequences below), and 2 U Go Taq flexi DNA polymerase (Promega Corp.,). RT-PCR was performed using the Eppendorf Realplex thermocycler with the following reaction conditions: 95°C for 10 min., 40 repeated cycles including denature (95°C), annealing (62°C), and extension (72°C), final extension for 5 min. at 72°C. PCR products were resolved on a 2% agarose gel and compared with a DNA ladder of known size (Fisher Scientific, Exactgene) to verify product size.

### Quantitative Polymerase Chain Reaction

qPCR was performed using FastStart DNA Master SYBR Green I according to manufacturer's instructions (Roche Molecular Biomedical, Indianapolis, IN). Master mix containing MgCL2, SYBR Green, and primer pairs (0.25 µM) were aliquotted into 96-well plates followed by the addition of 1/20^th^ of the reverse transcription reaction (cDNA). No template controls received DNA-free water of the same volume. All cDNA samples were tested in triplicate within an assay and each experiment was repeated three times. Real-time PCR reactions were carried out using the Eppendorf Realplex thermocycler with the following conditions: 95°C for 10 min., 40 repeated cycles including denature (95°C), annealing (60°C), and extension (72°C) with fluorescence detection at the end of each 72°C step, and then melted with continuous fluorescence detection to 95°C. PCR products were resolved on a 2% agarose gel and compared with a DNA ladder of known size (Fisher Scientific, Exactgene 50bp ladder) to confirm product size, and to verify specificity, the products were subjected to a thermal melting curve analysis to determine if the Tm of the product was consistent with the calculated theoretical Tm based on sequence. Primer sequences are as follows: **GnRH**: forward - 5′CTGCTGACTGTGTGTTTGGAAGG 3′; reverse – 5′CCTGGCTTCCTCTTCAATCA 3′. **FGFR1**: forward – 5′ATGGTTGACCGTTCTGGAAG 3′; reverse – TGGCTATGGAAGTCGCTCTT 3′; **FGFR3**: forward – 5′GAGACTTGGCTGCCAGAAAC 3′; reverse – 5′GGAGGACACCAAAAGACCA 3′. **FGF8**: forward – 5′ GAGCAACGGCAAAGGCAAGG 3′; reverse – 5′ CTCAACTACCCGCCCTTCAC 3′. The FGF sequence targets exon 5 which is present in all FGF8 splice variants.

All samples were first normalized to the constitutively expressed hypoxanthine phosphoribosyl transferase 1 (HPRT) housekeeping gene followed by absolute quantification extrapolated from known quantities in a standard curve. The Eppendorf Realplex software plots a standard curve of the crossing line intercepts of the standard vs. the known concentrations of these standards. The crossing line intercept is parallel to the x-axis on a graph of fluorescent intensity vs. cycle number and occurs at a point where the template amplification enters the logarithmic phase of the curve. Samples with higher concentrations of starting material enter the logarithmic phase earlier than samples with a lower concentration of starting material and consequently, have a smaller crossing point value. The crossing line intercept of unknowns is then compared with that of known values to calculate the actual amount. Data are represented as mRNA copies/µg total RNA.

### Statistics

Data were analyzed by one-way ANOVA followed by Tukey's HSD test. Differences were considered significant when P<0.05. All transfection data are represented as percent change compared to vehicle-treated, empty vector controls.
